# Olfactory neuroblastoma treated with minimally invasive surgery and adjuvant radiotherapy: a case report and review of the literature

**DOI:** 10.1259/bjrcr.20170077

**Published:** 2018-01-10

**Authors:** Domenico Cante, Cristina Piva, Piera Sciacero, Pierfrancesco Franco, Edoardo Petrucci, Valeria Casanova Borca, Fabrizio Marola, Libero Tubino, Giorgio Vellani, Maria Rosa La Porta

**Affiliations:** 1 Department of Radiation Oncology, Ivrea Community Hospital, Ivrea, Italy; 2 Department of Oncology, Radiation Oncology, University of Turin, Turin, Italy; 3 Department of Medical Physics, Ivrea Community Hospital, Ivrea, Italy; 4 Department of Otolaryngology, Chivasso Hospital, Chivasso, Italy; 5 Department of Medical Oncology, Chivasso Hospital, Chivasso, Italy

## Abstract

Olfactory neuroblastoma (ON) is a rare tumour of the olfactory neuroepithelium that is characterized by a pattern of slow growth and local recurrences. Combination of surgery and radiotherapy, with or without chemotherapy, is considered to be the standard of care for primary site disease. Recent literature supports the view that endoscopic resection followed by adjuvant radiotherapy correlates with better outcome. In this short communication, we present a case report of olfactory neuroblastoma arising in the right nasal sinus in a 34-year-old male. This patient was treated with endoscopic resection and external beam radiotherapy to the right nasal sinus with intensity-modulated radiation therapy (IMRT) technique. After 2 years follow-up, the patient is free of tumour without any late effect related to therapies. We believe that, in such patients, a treatment strategy including endoscopic resection followed by adjuvant radiotherapy may be effective and feasible and should be considered the gold standard of care.

## Introduction

Olfactory neuroblastoma (ON) originates from the olfactory epithelium. Unilateral nasal obstruction and epistaxis are the most common symptoms. Furthermore, headache, sinus pain, excessive lacrimation, rhinorrhea, anosmia and changes in vision may occur. Treatment modalities for ON are surgery combined with radiotherapy (RT) and/or chemotherapy.^1^ In this short communication, we report the case of a patient with a mass in the right nasal cavity who was treated by endoscopic resection and adjuvant RT.

## Case Report

**Figure 1.**
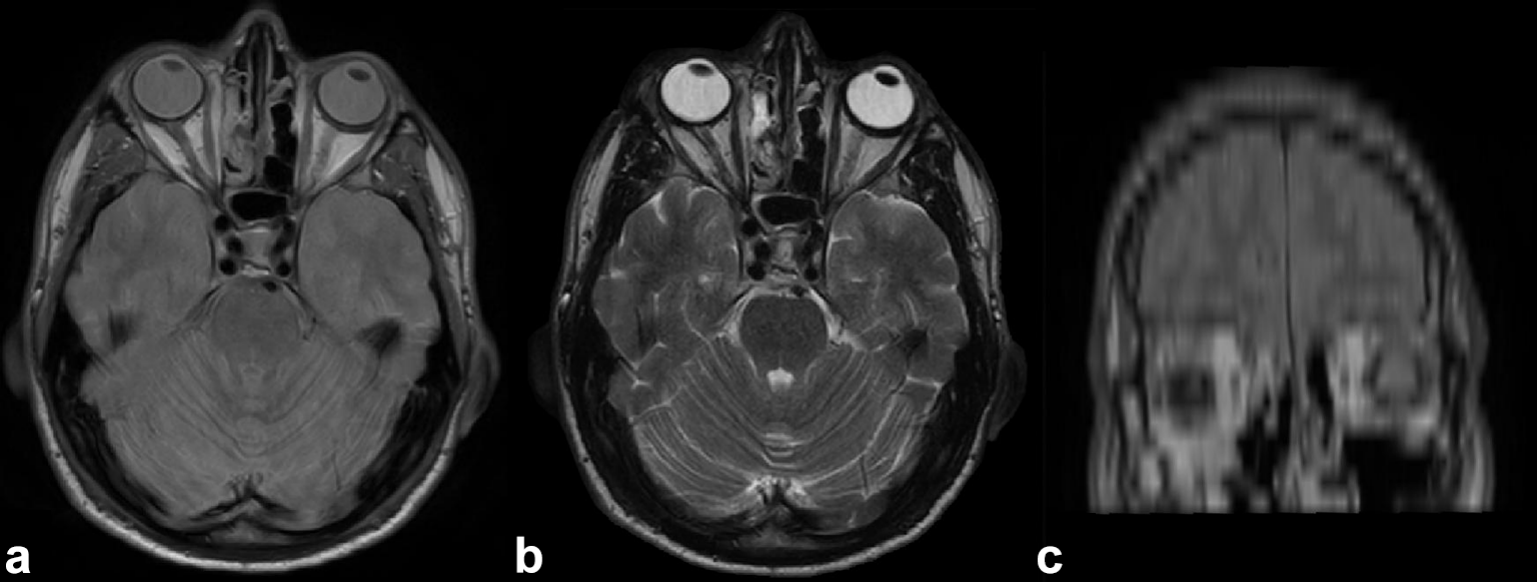
Hypointense *T*_1_ weighted (a), hyperintense *T*_2_ weighted (b) and coronal (c) preoperative MRI images

**Figure 2.**
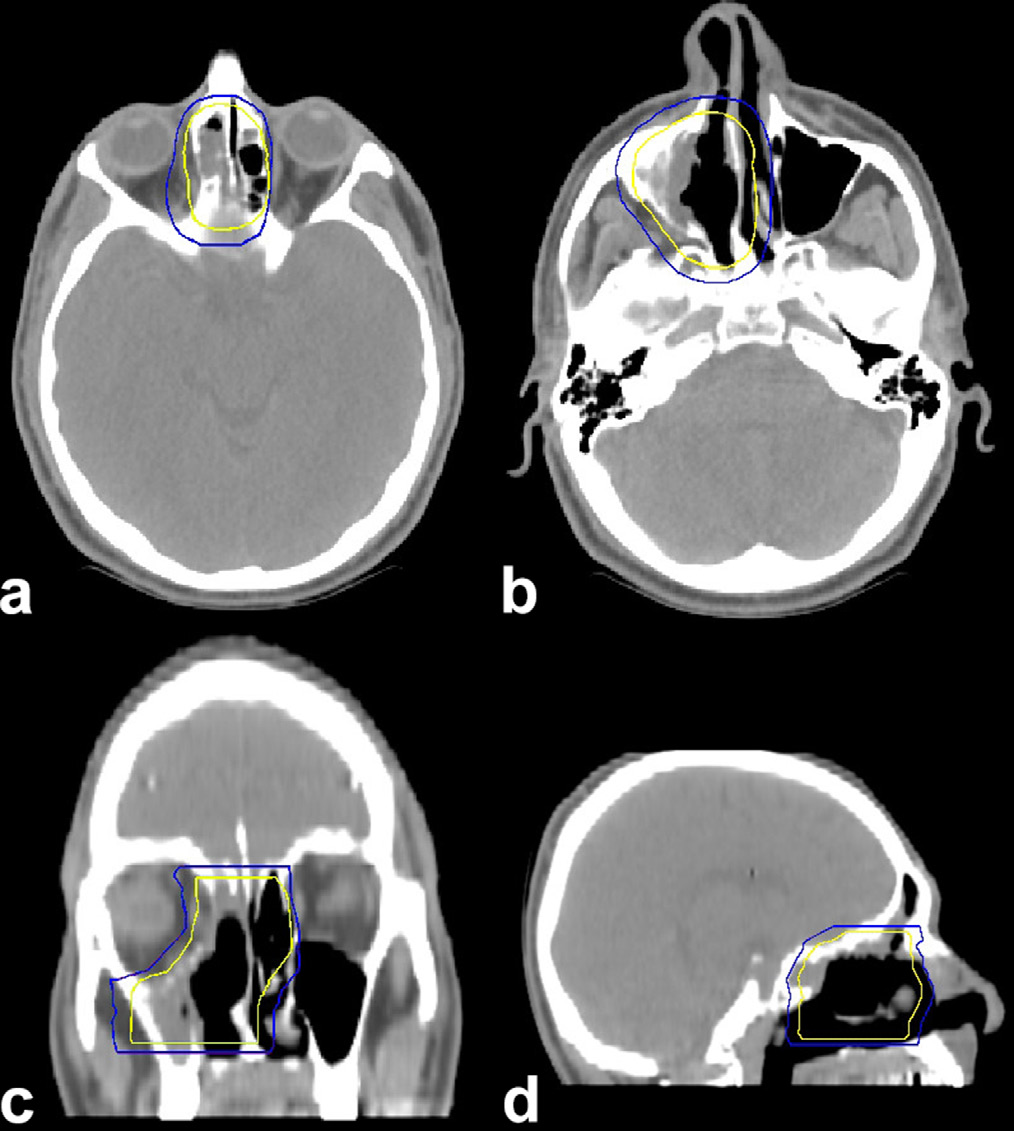
Axial (a, b), coronal (c) and sagittal (d) views of target volumes: inner lines correspond to clinical target volume, whereas outer lines represent planning target volume

**Figure 3.**
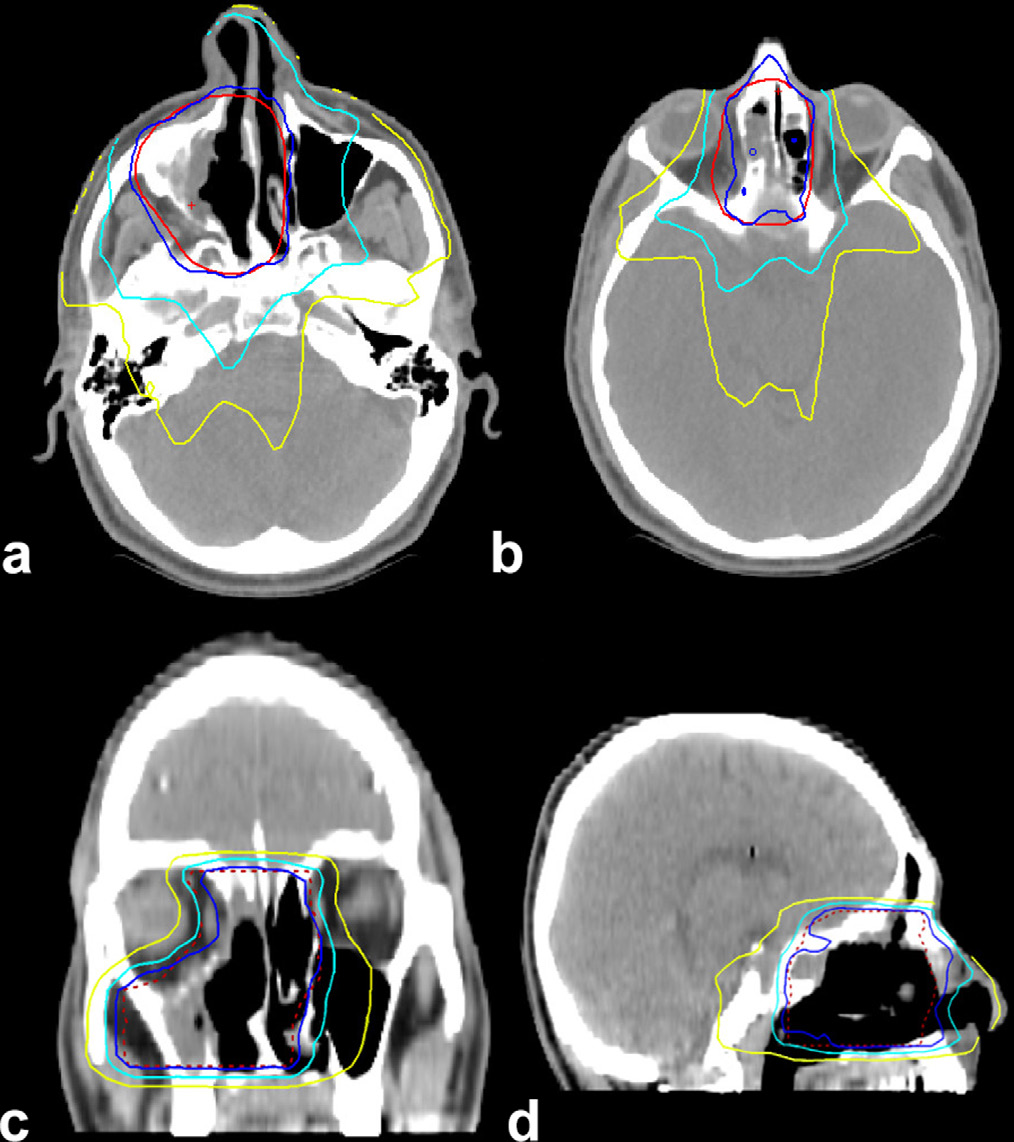
Dose distribution in axial (a, b), coronal (c) and sagittal (d) CT planning slices: the inner line corresponds to the planning target volume, whereas the other lines going outwards represent 95, 80 and 50% isodoses, respectively

A 34-year-old male was referred to our hospital where he presented a 6-month history of unilateral nasal obstruction and frontal headache. Endoscopic examination showed a polypoid mass in the right nasal cavity and permitted biopsy of the lesion. Histological findings documented an olfactory neuroblastoma of Grade II according to Hyams grading system. MRI was performed, revealing a well-circumscribed lesion in the right nasal sinus, hypointense on *T*_1_ weighted and hyperintense on *T*_2_ weighted sequences (Figure 1). This disease presentation corresponded to Kadish stage B. After discussion in the multidisciplinary tumour board, a bimodality therapeutic approach consisting of endoscopic resection followed by adjuvant radiotherapy (RT) was chosen. The patient was treated with a wide tumour excision by nasal endoscopic surgery, and postoperative pathology confirmed a moderate grade ON. 20 days later, the patient was planned to receive adjuvant external beam RT, delivered with a step and shoot (S &S) intensity-modulated radiation therapy (IMRT) technique. After proper immobilization (flat headboard and thermoplastic mask), a planning CT simulation with 3 mm slice thickness was performed. Target volume and organs at risk were contoured using the treatment planning system Masterplan, Oncentra (Nucleotron, Crawley, UK). A semi-automatic rigid registration between planning CT scan and diagnostic MRI images was performed to better define the clinical target volume (CTV) that included the preoperative tumour bed. A 5-mm isotropic margin was added to the CTV to obtain the planning target volume (PTV) (Figure 2). Beam geometry in IMRT plan consisted of five coplanar 6 MV fields. The prescribed dose was 60 Gy in 30 fractions (2 Gy daily) defined as the mean dose planned to the PTV with 95% of the PTV receiving ≥95% of the prescribed dose. Dose–volume histogram  was calculated for the IMRT plan for the following volumes: PTVs, spinal cord, brainstem, optic chiasm, eyes, optic nerves and lens. The dose–volume constraints were satisfied: 0.03 cc of the optic chiasm, optic nerves, eyes and brainstem should receive <54 Gy, spinal cord 45 Gy and lens 6 Gy. The dose distribution is shown in Figure 3. Radiation treatment was well tolerated with Grade 1 skin acute toxicity according to Radiation Therapy Oncology Group scale and nasal obstruction. No treatment interruption occurred. The patient is still under regular follow-up based on MRI and nasal endoscopy; after 2 years of observation, he continues to be free from disease without any late complications of therapy.

## Discussion

ON is a rare malignant tumour of the nasal cavity and it arises from the olfactory neuroepithelium located in the nasal septum.^2^ Commonly, this tumour causes unilateral nasal obstruction and epistaxis. Minor manifestations are anosmia, headache, sinus pain, rhinorrhea and epiphora. In the present case, the patient showed unilateral nasal obstruction and frontal headache. Clinically, ON is staged using the Kadish system that is based on the spread of the tumour.^3^ According to this system, stage A corresponds to tumours confined to the nasal cavity, stage B includes lesions involving also the paranasal sinuses, whereas stage C presents masses that extent beyond the nasal cavity and paranasal sinuses.^3^ MRI scan is essential to study disease extension and usually reveals a tumour mass presenting a low-intensity signal in *T*_1_ weighted images and an iso- or high-intensity signal in *T*_2_ weighted images. A key issue consists in early histological diagnosis of ON through endoscopic biopsy. Many studies tend to divide ON into low-grade and high-grade lesions according to Hyams classification identifying two distinct entities. Malouf et al^4^ showed that patients with high-grade ON had larger tumours, frequent lymph node involvement and more often leptomeningeal metastasis compared to low-grade ON. In our case, MRI showed a Kadish stage B ON and endoscopic biopsy revealed a low-grade ON.

The available literature indicates that a combination of surgery and RT is the best treatment approach.^5^ Although craniofacial resection is considered the gold standard surgical treatment, some recent reports suggest treating ON with minimally invasive surgery. In fact, endoscopic approaches present some advantages such as shorter surgical time and hospitalization and a better quality of life.^6^ Table 1 reports studies including treatment characteristics and outcome for olfactory neuroblastoma. Some reports showed that the addition of postoperative radiation to surgery significantly improves local control rates. In the study of Dulguerov and Calcaterra^7^ local control was 86% with combined treatment and 17% with surgery alone. Morita et al^6^ reported a local recurrence rate of 55% in patients who underwent total resection alone versus 19% in patients treated with total resection and adjuvant RT. Chao et al^12^ recommended a combined modality treatment in all Kadish stages of disease. In the study of Diaz et al^3^ 10-year disease-specific survival rate was 100% in patients with Kadish A/B stage who underwent surgery and postoperative RT. More recently, Ow et al^23^ retrospectively reviewed 70 patients affected by ON treated at the MD Anderson Cancer Center showing a median disease-specific survival of 87.9 months for patients who received surgery alone and 218.5 months for those underwent surgery and postoperative RT (*p* = 0.047). They concluded that survival is considerably better when surgical resection is followed by adjuvant RT. In the study of Mori et al^26^ multimodal therapy including RT with precise treatment planning based on CT simulation achieved an excellent local control rate, and the 5-year overall survival (OS) and relapse-free survival (RFS) rates were estimated at 88 and 74%, respectively.

**Table 1.** Studies reporting treatment characteristics and outcome for olfactory neuroblastoma

**Study, year**	**Period**	**Patients (*n*)**	**Treatment**	**RT technique**	**Mean Dose (Gy)**	**Follow-up (months)**	**Median Survival (months)**	**5-year OS**	**Other Survival**
Dulguerov and Calcaterra, 1992^7^	1970–1990	24	S only RT only S + RT± CT CT + RT	2D-RT 3D-CRT	60	–	–	–	5-year DSS 74% 5-year RFS 58%
Polin et al, 1998^8^	1976–1994	34	RT ± CT + S	–	50.6	–	71	81	–
Resto et al, 2000^9^	1981–1998	27	S only RT only S + RT± CT	–	61.8	–	71	–	–
Eich et al, 2001^10^	1981–1998	17	RT only S + RT	2D-RT 3D-CRT	57.3	86	94	–	–
Simon et al, 2001^11^	1978–1998	13	S only RT only S + RT± CT	–	59.4	75	60	61	5-year DFS 56%
Chao et al, 2001^12^	1976–1996	25	S only RT ± CT S + RT± CT	2D-RT 3D-CRT	56.4	96	–	66.3	5-year DFS 56.3%
Gruber et al, 2002^13^	1980–2001	28	RT ± CT S + RT± CT	2D-RT 3D-CRT	60	68	–	–	5-year LPFS 81% 5 year DFS 70% 5-year CSS 77%
Argiris et al, 2003^14^	1981–2000	16	S ± CT S + RT± CT	–	55	51	60	60	5-year DFS 33%
Diaz et al, 2005^3^	1979–2002	30	S only S + RT RT ± CT	–	59.4	72	–	89	5-year RFS 69%
Castelnuovo et al, 2007^15^	1999–2004	10	S only S + RT	3D-CRT	56.1	37	37	–	–
Bachar et al, 2008^16^	1972–2006	39	S only RT only S + RT± CT	3D-CRT IMRT	53.13	–	140	87.9	5-year RFS 76% 5-year LRFS 82% 5-year LRRFS 82.5%
Ozsahin et al, 2010^17^	1971–2004	77	S only S + RT± CT RT ± CT	2D-RT 3D-CRT IMRT	60	72	–	64	5-year DFS 57%
Platek et al, 2011^18^	1973–2006	511	S only RT only S + RT Neither S nor RT	–	–	–	–	73 S + RT 68 s only 35 RT only 26 neither S nor RT	–
Back et al, 2012^19^	1990–2009	17	S only S + RT± CT RT ± CT	2D-RT 3D-CRT IMRT	60	57.5	60	68	5-year DFS 62%
Michel et al, 2012^20^	1978–2006	11	S only S + RT RT + CT	–	–	–	–	90	5-year DFS 54.5%
Modesto et al, 2013^21^	1998–2010	43	Multimodal therapy	3D-CRT IMRT	64	77	–	65	5-year PFS 57%
Kumar et al, 2013^22^	2006–2010	15	S + RT± CT RT ± CT	2D-RT 3D-CRT IMRT	54	23	35	45 (4 year)	4-year LRC 25%
Ow et al, 2014^23^	1992–2007	70	S only S + RT± CT	–	–	91.4	126.3	90	5-year DSS 90%
Rimmer et al, 2014^24^	1978–2013	95	S only S + RT± CT	2D-RT 3D-CRT IMRT	–	88.6	224	83.4	5-year DFS 80%
Feng et al, 2015^25^	2001–2012	24	S only S + RT± CT	–	60	44	–	82 (3 year)	3-year DFS 70.8%
Mori et al, 2015^26^	1992–2013	17	S + RT Multimodal therapy	3D-CRT IMRT	–	95	–	88	5-year RFS 74%
Lapierre et al, 2016^27^	1993–2015	10	S only S + RT± CT	3D-CRT IMRT	61	136	–	90 (10 year)	5-year PFS 70%

2D-RT, 2-dimensional radiotherapy; 3D-CRT, 3-dimensional conformal radiotherapy; CSS, cancer-specific survival; CT, chemotherapy; DFS, disease-free survival; DSS, disease-specific survival; IMRT, intensity-modulated radiation therapy; LPFS, local progression-free survival; LRC, locoregional control; LRFS, local relapse-free survival; LRRFS, locoregional relapse-free survival; OS, overall survival; PFS, progression-free survival; RFS, relapse-free survival; RT, radiotherapy; S, surgery.

With regard to the RT technique, when the tumour involves adjacent structures such as the infraorbital canal or optic nerve, IMRT is recommended because it better preserves closer structures.^23,  26, 27^ In our clinical case, a combined strategy consisting of endoscopic surgery and adjuvant IMRT was chosen.

The radiation dose greatly varies among the studies in the literature. In the postoperative setting, mean radiation doses of 56.9 Gy (range 50.0–67.2 Gy) and 54.57 Gy (range 45–60 Gy) were reported by Diaz et al^3^ and Bachar et al^16^ respectively. More recently, Mori et al used 50 to 66 Gy as postoperative RT, 40 Gy as preoperative RT and 54 to 66 Gy in the setting of sequential chemoradiation therapy. In the absence of randomized studies, Lapierre et al^27^ recommended the doses currently used in the treatment of other sinonasal tumours, between 60 and 70 Gy in 30–35 fractions based on tumour site, pathological characteristics and quality of surgical resection. In our clinical case, we delivered a total dose of 60 Gy in 30 fractions (2 Gy daily) because this is the schedule we used in the postoperative setting for sinonasal tumours and because the patient underwent a wide tumour excision by nasal endoscopic surgery with negative margins.

In patients affected with ON, it is difficult to decide the optimal RT treatment volume. Radiation fields should include the tumour bed and potential sites of local spread. Klepin et al^28^ suggested that the treatment volumes covered the entire nasal fossa, the maxillary sinuses with an extension into the ethmoid, the sphenoid sinus and the anterior cranial fossa to control brain invasion. The role of elective nodal irradiation (ENI) in ON is still controversial; the study of Elkon et al^29^ reported a neck nodal failure rate of 10% suggesting that ENI was not necessary in node-negative patients. More recently, other studies reported regional failure rates of 23.4 and 27% suggesting that ENI should be considered when the primary disease is locally advanced or when regional neck node is positive at diagnosis.^30,  31^ In 2011, Noh et al^32^ concluded that ENI for ON plays a limited role in preventing cervical nodal failure and that omitting ENI may be an option in patients affected by advanced disease treated with a combination of RT and chemotherapy. In the study of Lapierre et al^27^ none of the patients received prophylactic cervical irradiation and there were two recurrences (28%) in the neck nodes. Relapses were treated by surgery in one patient and by the combination of surgery and adjuvant RT in the other patient. In a recent large cohort study, a significant improvement in the 5-year local control rate with prophylactic nodal irradiation was demonstrated (75* vs *98% without and with ENI, respectively, *p* = 0.005). The authors concluded that ENI should be recommended as a part of the initial treatment strategy for patients staged with modified Kadish B/C.^33^

In our clinical case, after discussing with the patient about the literature data regarding the prophylactic cervical irradiation, the pros and cons and the side effects, we opted for an RT volume that included the tumour bed and we decided not to perform ENI owing to the limited Kadish B stage, radical surgery, absence of clinically and radiologically positive nodes and the possibility of treatment at the time of recurrence.

Furthermore, in addition to surgery and RT, chemotherapy may offer improvement in local control and reduction in the frequency of distant metastasis, especially in patients with unresectable tumours or in case of advanced disease and recurrent and metastatic lesions.^34^

## Conclusions

In our opinion, this case report shows that a combined modality approach with minimally invasive surgery and postoperative IMRT can be effective in this setting.^35^ Two years after treatment, there is no local recurrence in the nasal cavity nor late effects. Nevertheless, the possibility of late relapse requires an extended follow-up time.

## Learning points

1. In this case report of a rare clinicopathological entity, we showed the impact of bimodal therapy with minimally invasive surgery and adjuvant RT. This strategy has proved to be successful, representing a proof of principle for potential future studies.
2. Minimally invasive surgery is potentially feasible in olfactory neuroblastoma.
3. Adjuvant radiotherapy increases local control.
4. High-tech radiation provides a good balance between tumor control and normal tissue sparing.
5. Combination therapy is safe and effective in thi setting.

## Consent

Written informed consent for the case to be published (including images, case history and data) was obtained from the patient(s) for publication of this case report, including accompanying images.
